# Supplementary material for: Canonical Wnt Signaling Promotes Formation of Somatic Permeability Barrier for Proper Germ Cell Differentiation
Source: Front Cell Dev Biol. 2022 Apr 19;10:877047. doi: 10.3389/fcell.2022.877047 (PMC9062081; doi:10.3389/fcell.2022.877047)
Supplement: Supplementary file 1 [file Table1.docx]

| **Supplementary Table 1. Expression patterns of the somatic GAL4 drivers used in this study** | | | |
| --- | --- | --- | --- |
| GAL4 | LL3 gonad | Pupal germarium | Adult germarium |
| *tj-GAL4* | ICs^[1, 2]^ | - | ECs, FCs^[2, 3]^ |
| *c587-GAL4* | Most somatic cells^[2]^ | Gradually enriched to ECs at late pupal stage^[2]^ | ECs^[2, 3]^ |
| *bab1-GAL4* | Most somatic cells^[2]^ | Gradually enriched to TF and cap cell at late pupal stage^[2]^ | TF, cap cells, and anterior ECs^[2, 3]^ |

ICs, intermingled cells; ECs, escort cells; FCs, follicle cells; TF, terminal filament.

1. Lai CM, Lin KY, Kao SH, Chen YN, Huang F, Hsu HJ. Hedgehog signaling establishes precursors for germline stem cell niches by regulating cell adhesion. J Cell Biol. 2017;216(5):1439-53. doi: 10.1083/jcb.201610063. PubMed PMID: 28363970; PubMed Central PMCID: PMCPMC5412570.

2. Tseng CY, Su YH, Yang SM, Lin KY, Lai CM, Rastegari E, et al. Smad-Independent BMP Signaling in Somatic Cells Limits the Size of the Germline Stem Cell Pool. Stem Cell Reports. 2018;11(3):811-27. doi: 10.1016/j.stemcr.2018.07.008. PubMed PMID: 30122445; PubMed Central PMCID: PMCPMC6135924.

3. Weaver LN, Ma T, Drummond-Barbosa D. Analysis of Gal4 Expression Patterns in Adult Drosophila Females. G3 (Bethesda). 2020;10(11):4147-58. doi: 10.1534/g3.120.401676. PubMed PMID: 32917721.
