# Supplementary material for: Canonical Wnt Signaling Promotes Formation of Somatic Permeability Barrier for Proper Germ Cell Differentiation
Source: Front Cell Dev Biol. 2022 Apr 19;10:877047. doi: 10.3389/fcell.2022.877047 (PMC9062081; doi:10.3389/fcell.2022.877047)
Supplement: Supplementary file 2 [file Presentation1.PPTX]

## Slide 1
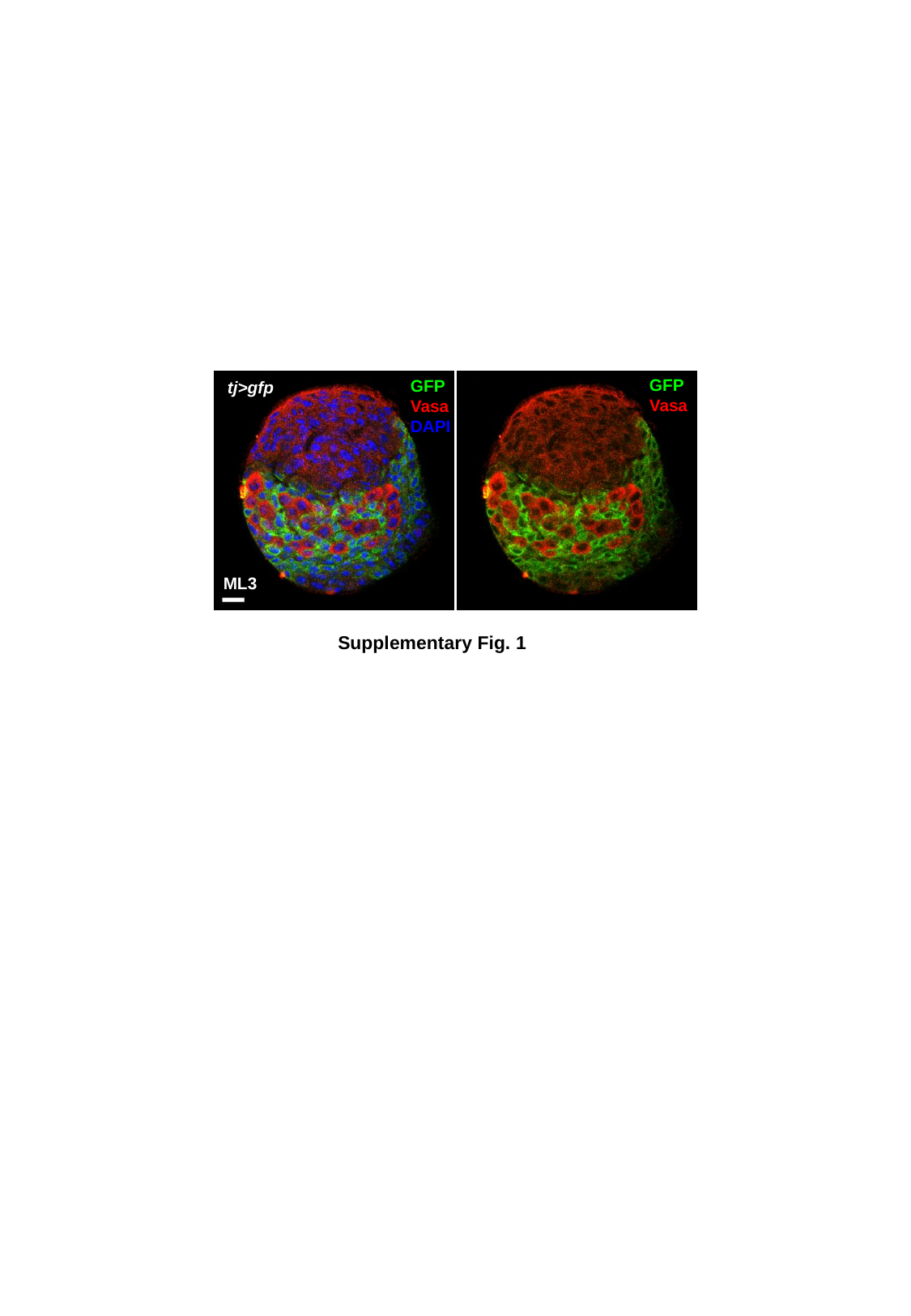

GFP Vasa
GFP Vasa DAPI
tj>gfp
ML3
Supplementary Fig. 1

## Slide 2
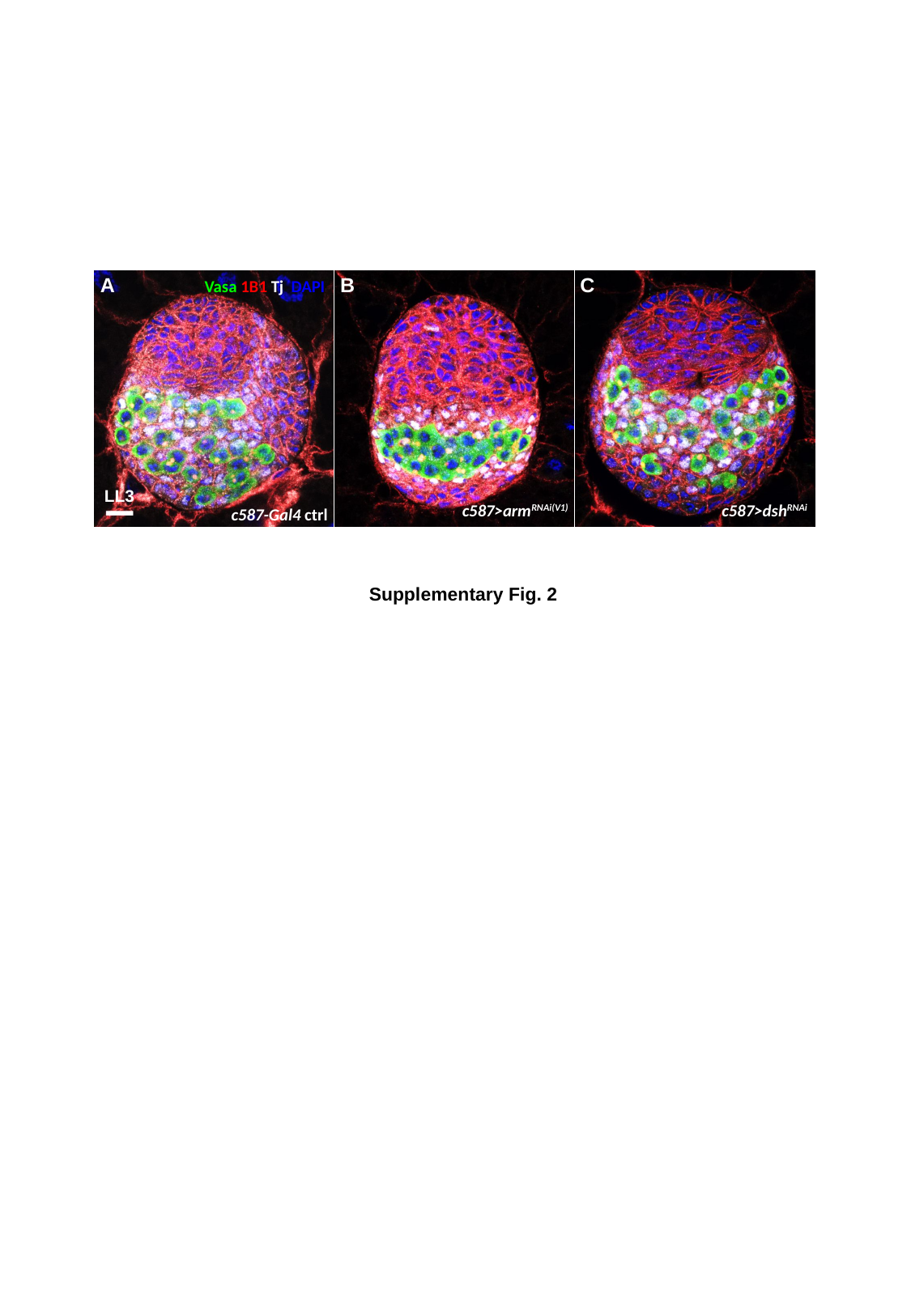

A
B
C
Vasa 1B1 Tj DAPI
LL3
c587>armRNAi(V1)
c587>dshRNAi
c587-Gal4 ctrl
Supplementary Fig. 2
Embryo-LL3 KD

## Slide 3
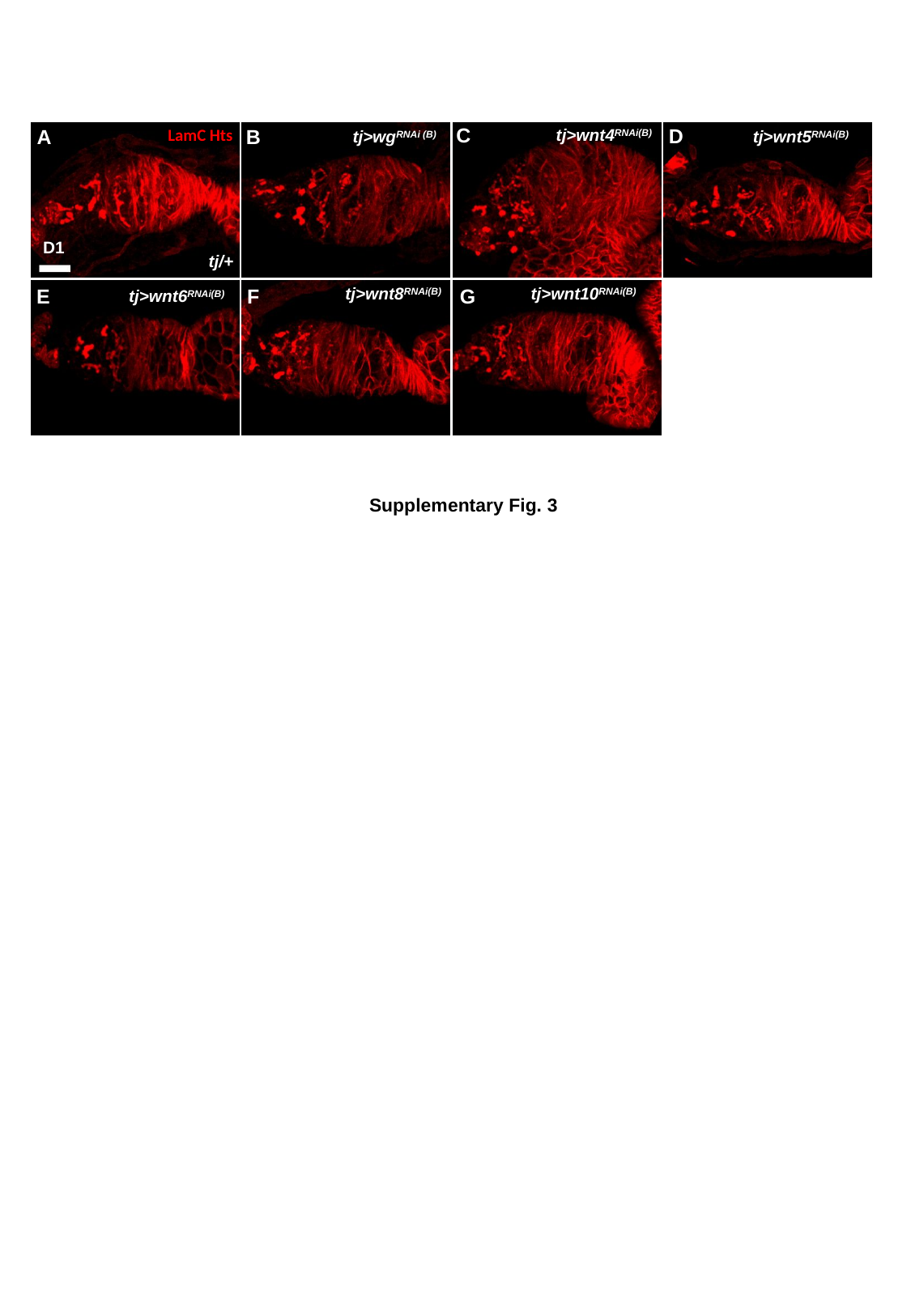

C
D
A
B
 LamC Hts
tj>wnt4RNAi(B)
tj>wgRNAi (B)
tj>wnt5RNAi(B)
D1
tj/+
tj>wnt10RNAi(B)
tj>wnt8RNAi(B)
G
F
E
tj>wnt6RNAi(B)
Supplementary Fig. 3

## Slide 4
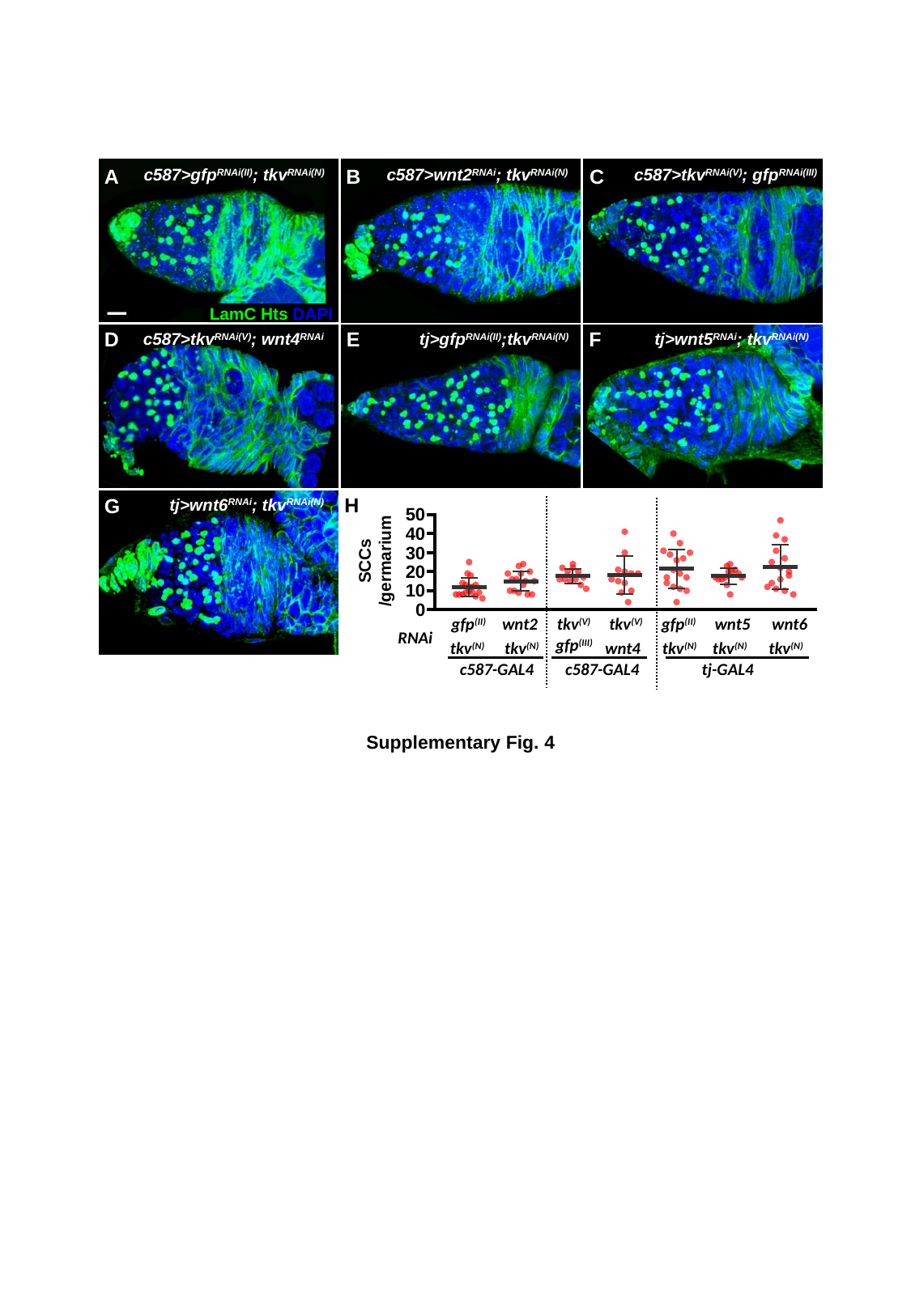

A
B
C
c587>gfpRNAi(II); tkvRNAi(N)
c587>wnt2RNAi; tkvRNAi(N)
c587>tkvRNAi(V); gfpRNAi(III)
Whole stage KD
LamC Hts DAPI
D
E
F
c587>tkvRNAi(V); wnt4RNAi
tj>gfpRNAi(II);tkvRNAi(N)
tj>wnt5RNAi; tkvRNAi(N)
H
G
tj>wnt6RNAi; tkvRNAi(N)
SCCs
/germarium
wnt6
gfp(II)
wnt5
wnt2
tkv(V)
tkv(V)
gfp(II)
RNAi
gfp(III)
tkv(N)
tkv(N)
wnt4
tkv(N)
tkv(N)
tkv(N)
tj-GAL4
c587-GAL4
c587-GAL4
Supplementary Fig. 4

## Slide 5
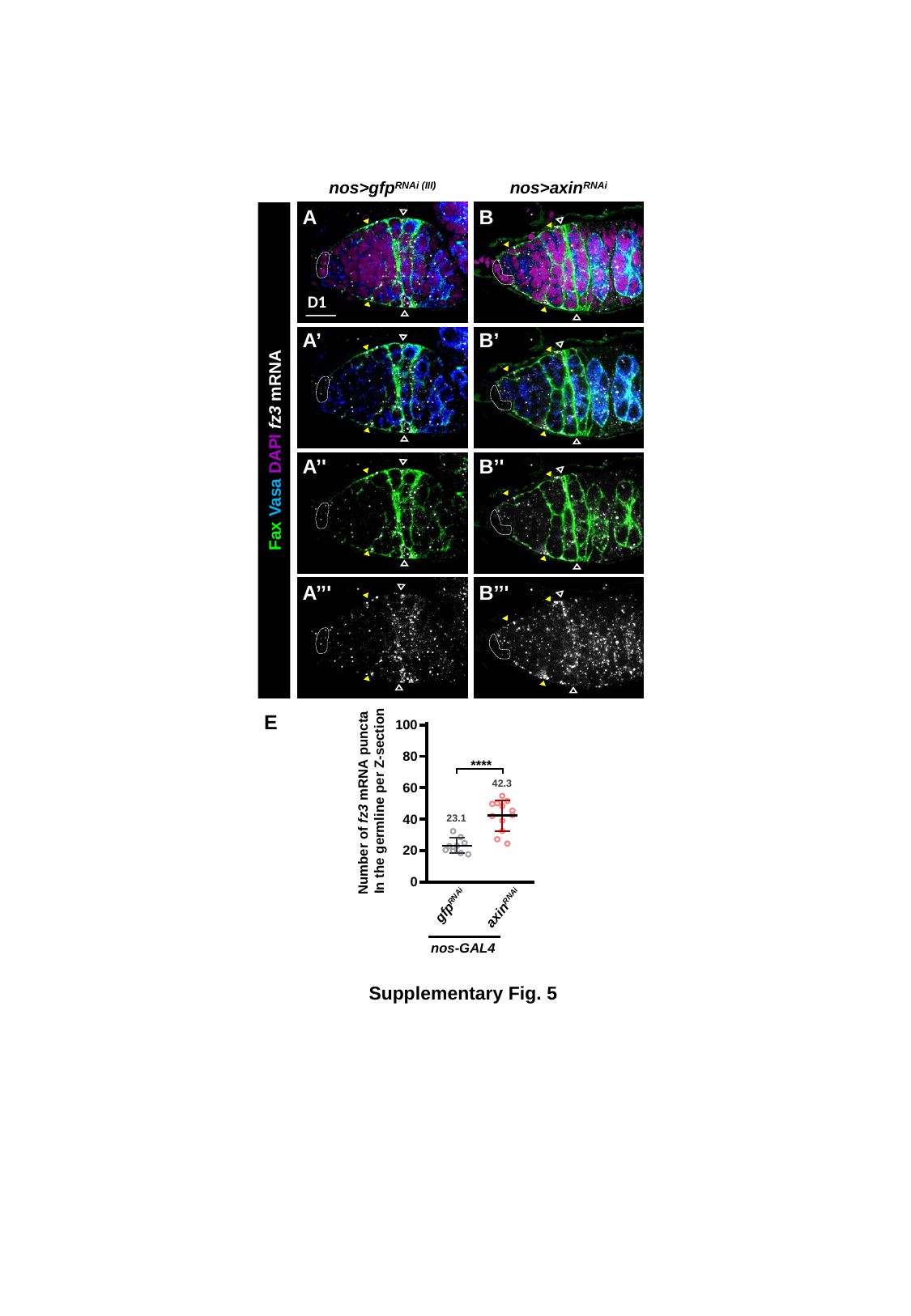

nos>gfpRNAi (III)
nos>axinRNAi
A
A’
A’'
A’’'
B
B’
B’'
B’’'
D1
Fax Vasa DAPI fz3 mRNA
****
42.3
Number of fz3 mRNA puncta
In the germline per Z-section
23.1
gfpRNAi
axinRNAi
nos-GAL4
E
Supplementary Fig. 5

## Slide 6
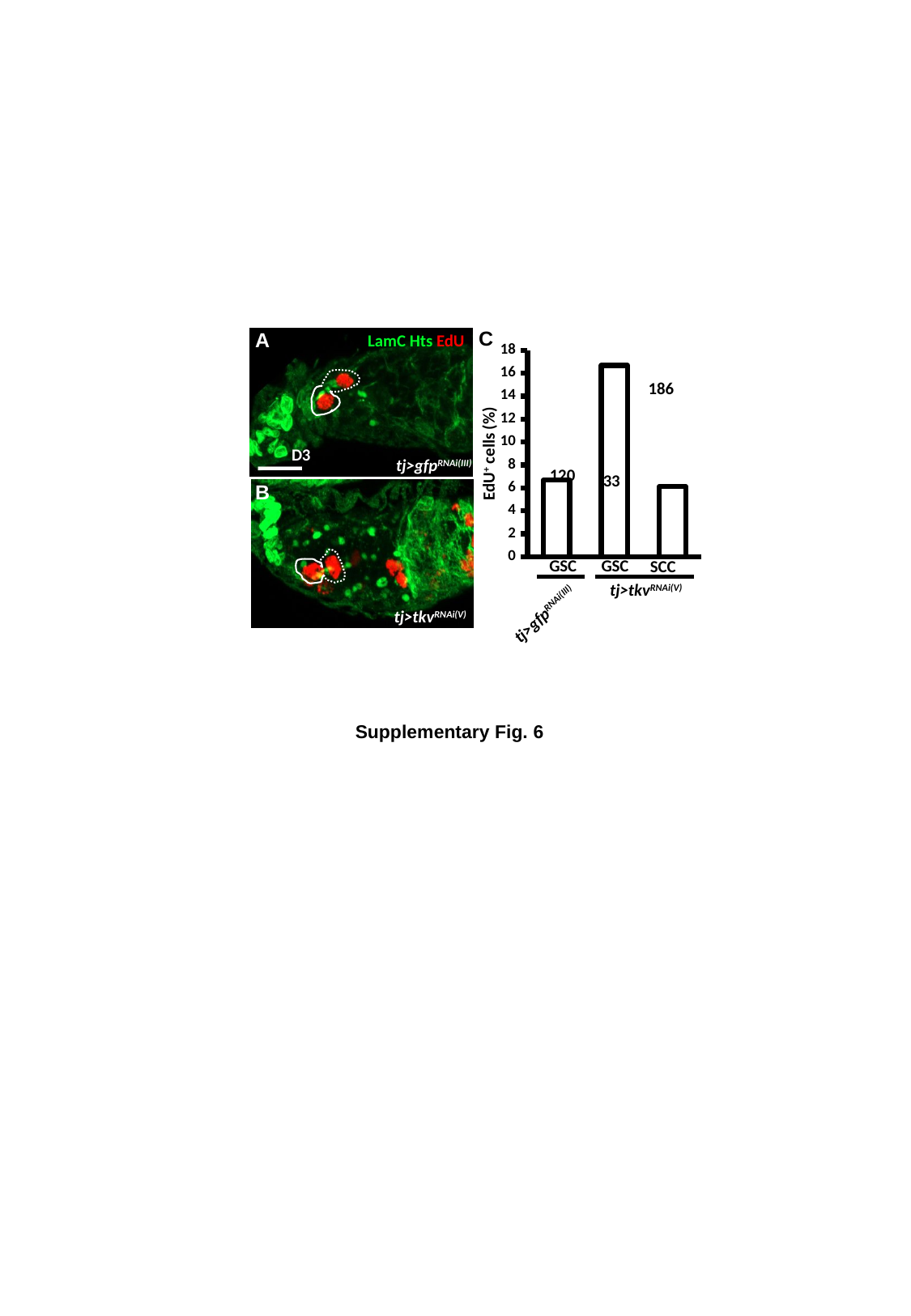

C
A
LamC Hts EdU
A
D3
tj>gfpRNAi(III)
B
B
### Chart
| Category | |
|---|---|
| Edu+ GSC | 6.67 |
| Edu+ SCC | 16.67 |
| Edu+ GSC | 6.1 |
186
EdU+ cells (%)
120
33
tj>tkvRNAi(V) ; gfpRNAi(III)
GSC
GSC
SCC
tj>tkvRNAi(V)
tj>tkvRNAi(V)
tj>gfpRNAi(III)
Supplementary Fig. 6

## Slide 7
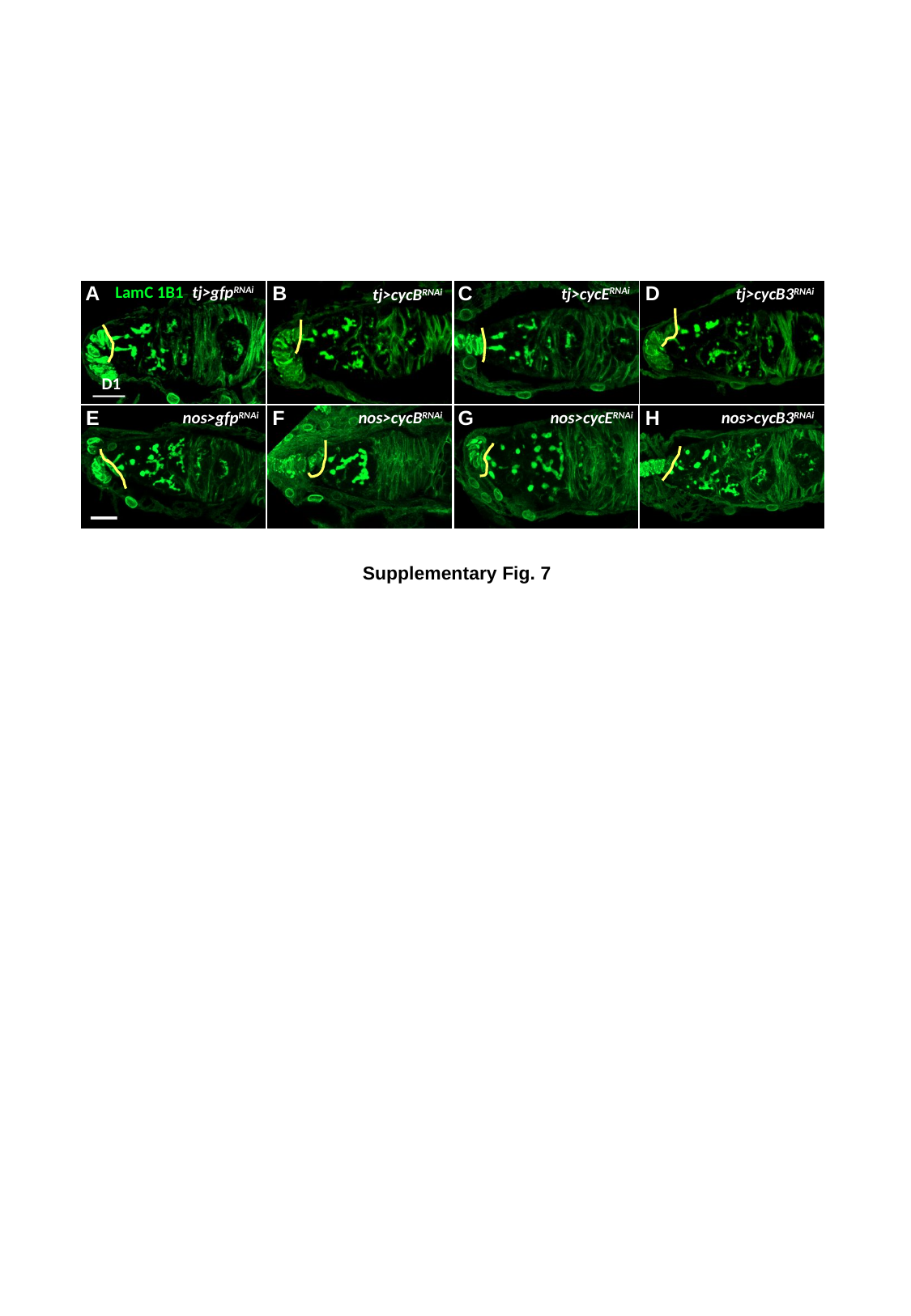

A
B
C
D
LamC 1B1
tj>gfpRNAi
tj>cycERNAi
tj>cycB3RNAi
tj>cycBRNAi
D1
E
F
G
H
nos>gfpRNAi
nos>cycBRNAi
nos>cycERNAi
nos>cycB3RNAi
Supplementary Fig. 7

## Slide 8
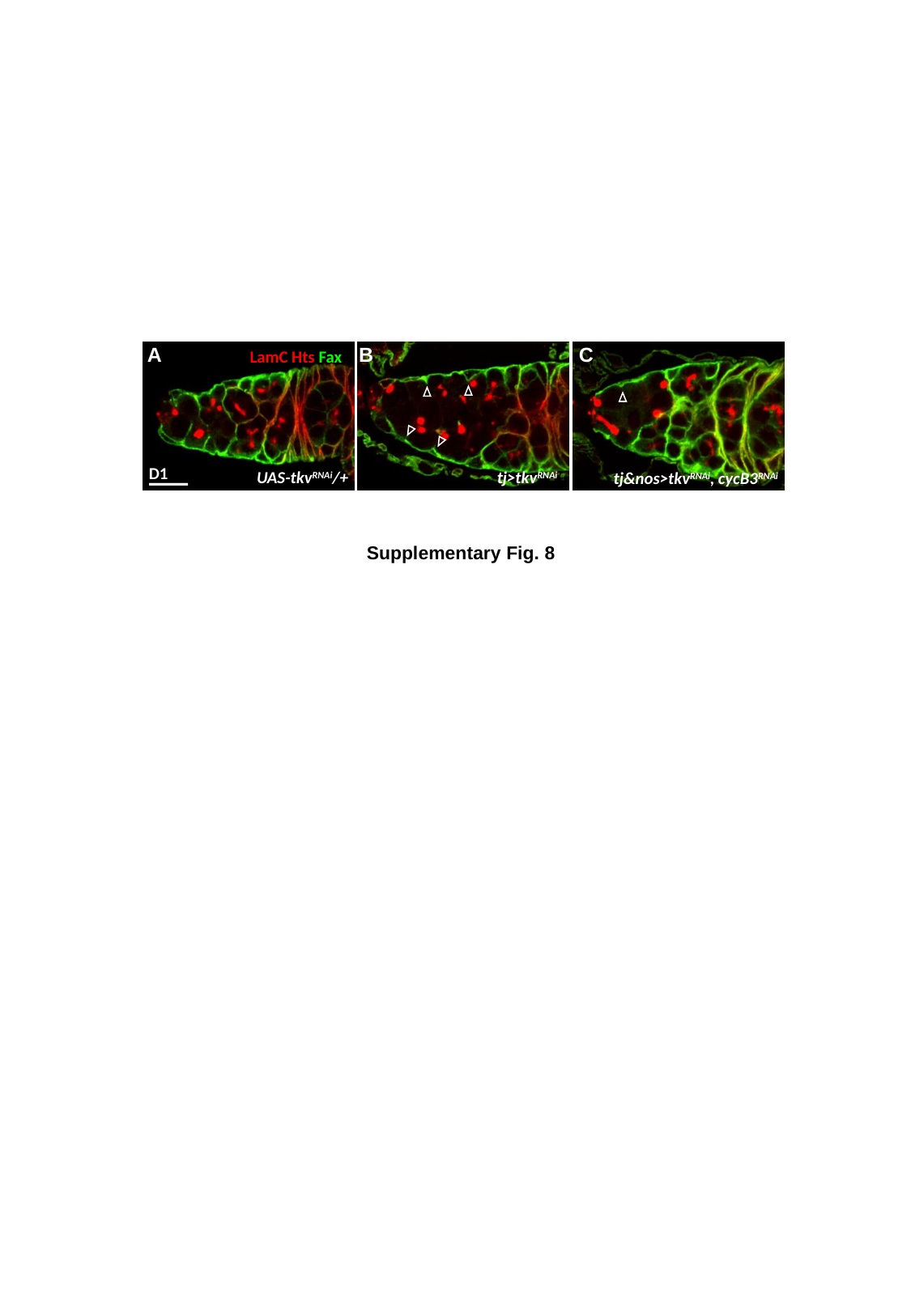

A
B
C
LamC Hts Fax
UAS-tkvRNAi/+
tj>tkvRNAi
tj&nos>tkvRNAi, cycB3RNAi
D1
Supplementary Fig. 8

## Slide 9
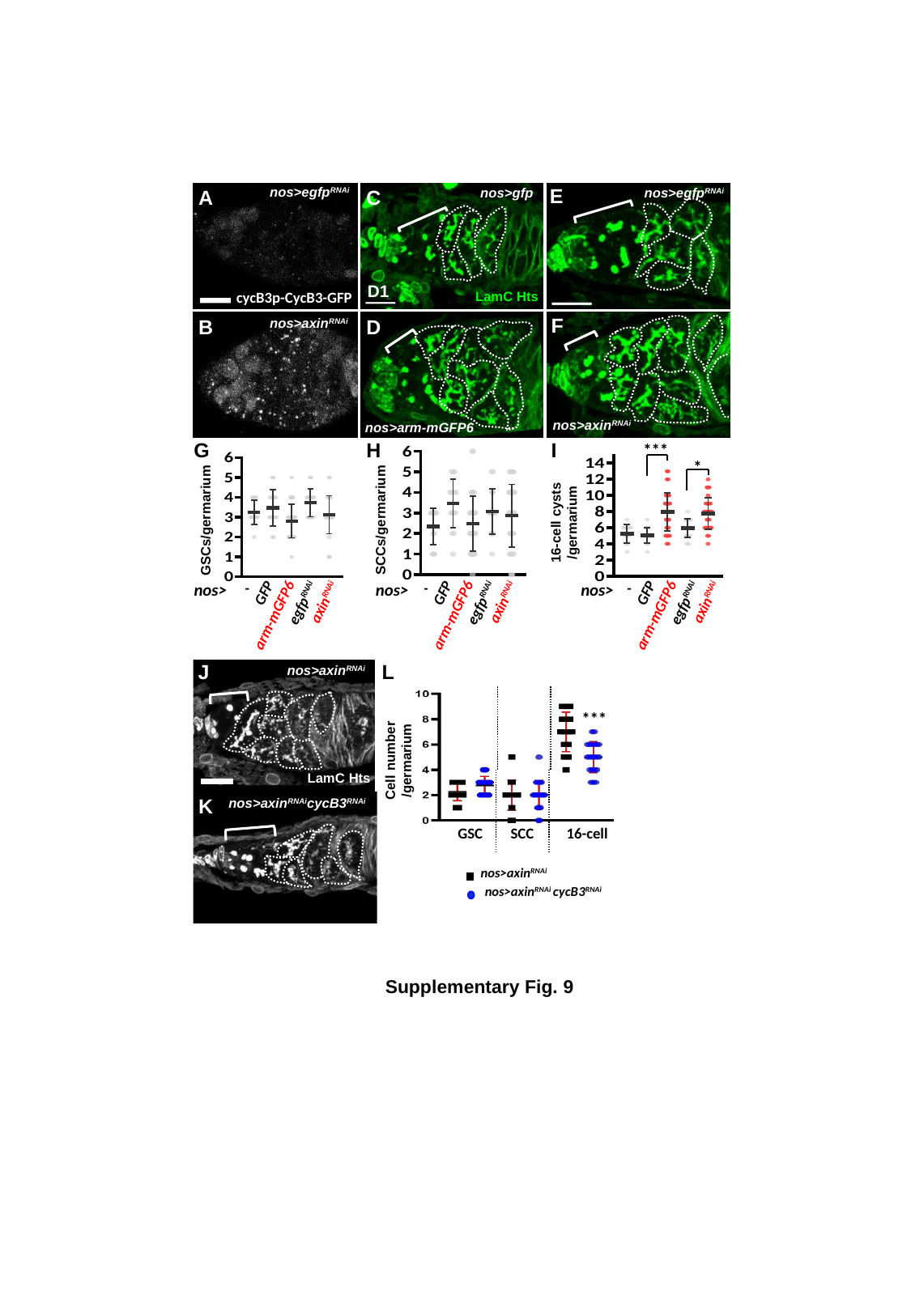

nos>egfpRNAi
A
cycB3p-CycB3-GFP
B
nos>axinRNAi
E
C
nos>gfp
nos>egfpRNAi
D1
LamC Hts
F
D
nos>axinRNAi
nos>arm-mGFP6
G
H
I
***
*
16-cell cysts
/germarium
SCCs/germarium
GSCs/germarium
-
arm-mGFP6
egfpRNAi
axinRNAi
GFP
-
arm-mGFP6
egfpRNAi
axinRNAi
GFP
-
arm-mGFP6
egfpRNAi
axinRNAi
GFP
nos>
nos>
nos>
J
L
nos>axinRNAi
***
GSC
SCC
16-cell
Cell number
/germarium
LamC Hts
K
nos>axinRNAicycB3RNAi
nos>axinRNAi
nos>axinRNAi cycB3RNAi
Supplementary Fig. 9

## Slide 10
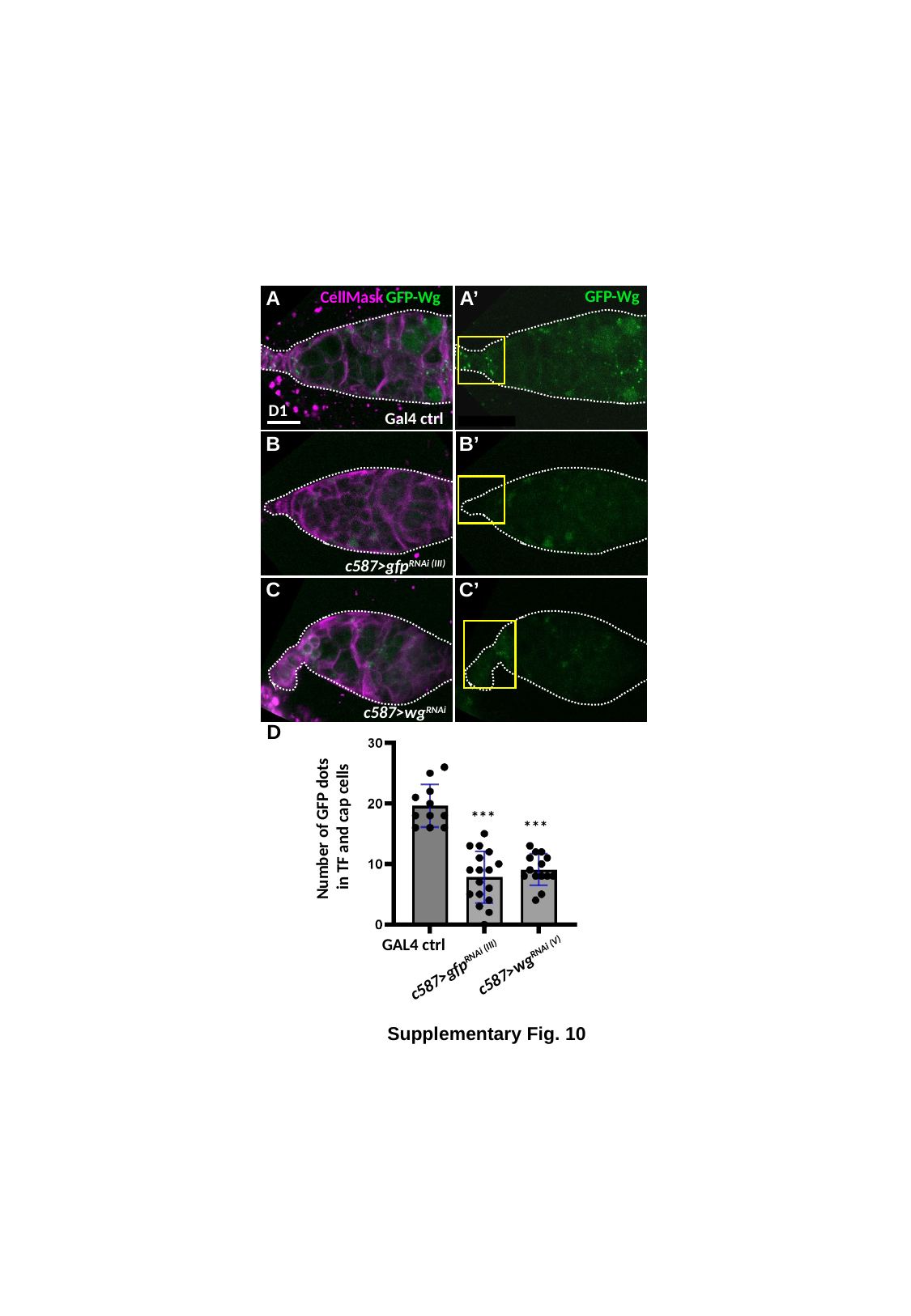

Gal4 ctrl #1
GFP-Wg
A
A’
CellMask GFP-Wg
D1
Gal4 ctrl
B
B’
c587>gfpRNAi (III)
C
C’
c587>wgRNAi
D
Number of GFP dots
in TF and cap cells
***
***
GAL4 ctrl
c587>wgRNAi (V)
c587>gfpRNAi (III)
Supplementary Fig. 10

## Slide 11
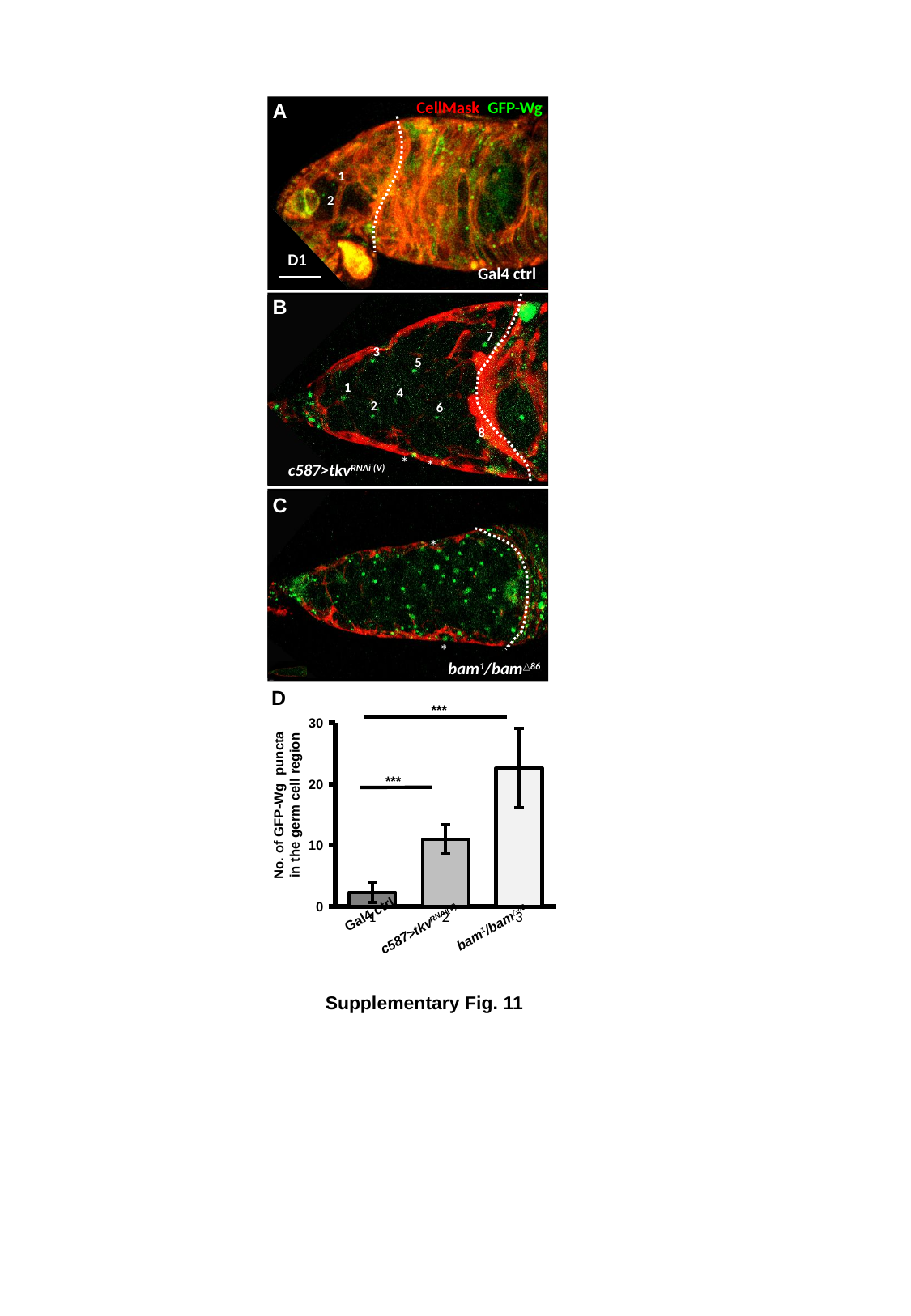

CellMask
GFP-Wg
A
1
2
D1
Gal4 ctrl
B
7
3
5
1
4
2
6
8
*
*
c587>tkvRNAi (V)
C
*
*
bam1/bam△86
D
***
### Chart
| Category | |
|---|---|***
No. of GFP-Wg puncta
in the germ cell region
Gal4 ctrl
bam1/bam△86
c587>tkvRNAi(V)
Supplementary Fig. 11

## Slide 12
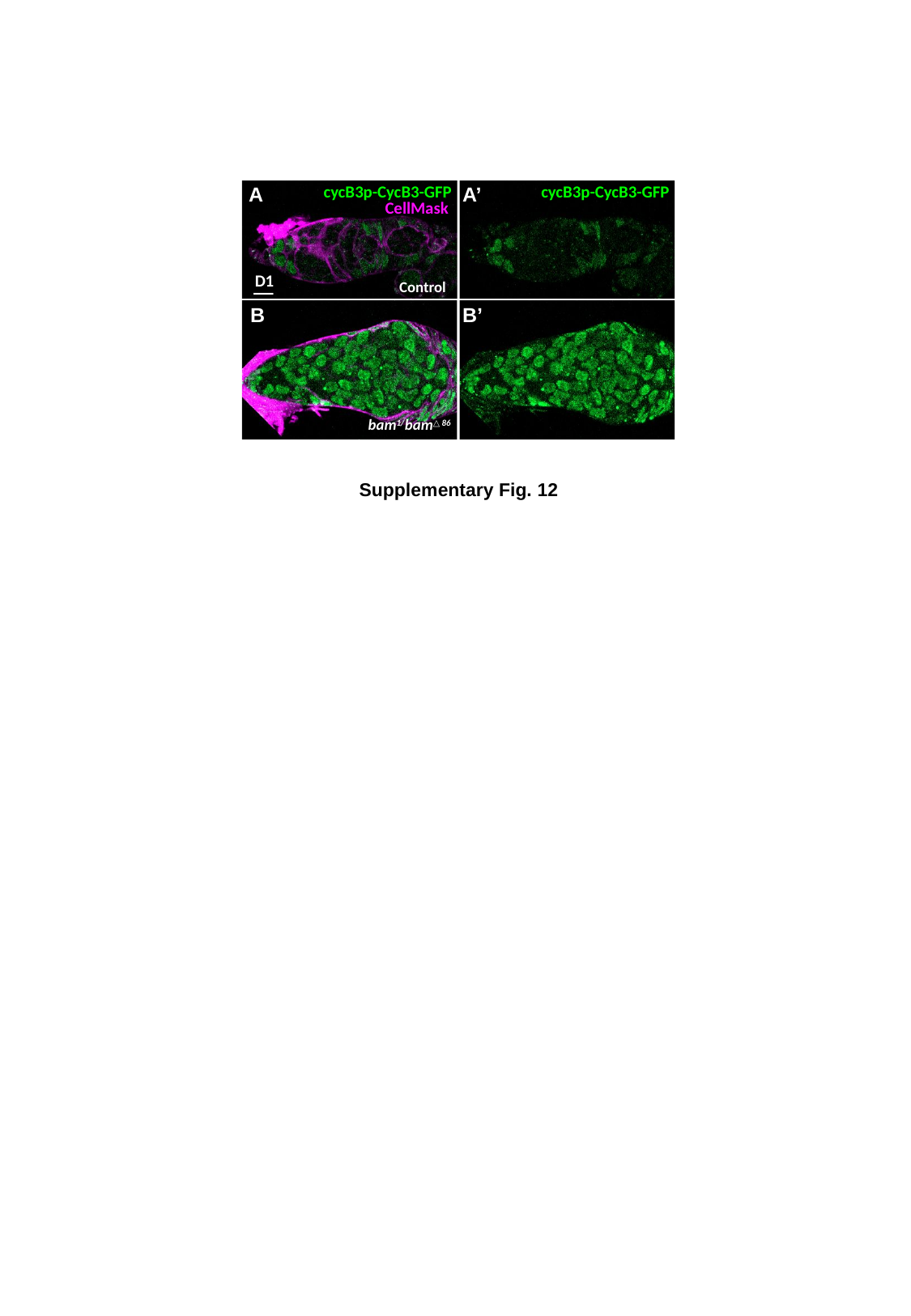

cycB3p-CycB3-GFP
cycB3p-CycB3-GFP
A
A’
 CellMask
D1
B
B’
bam1/bam△ 86
Control
Supplementary Fig. 12

## Slide 13
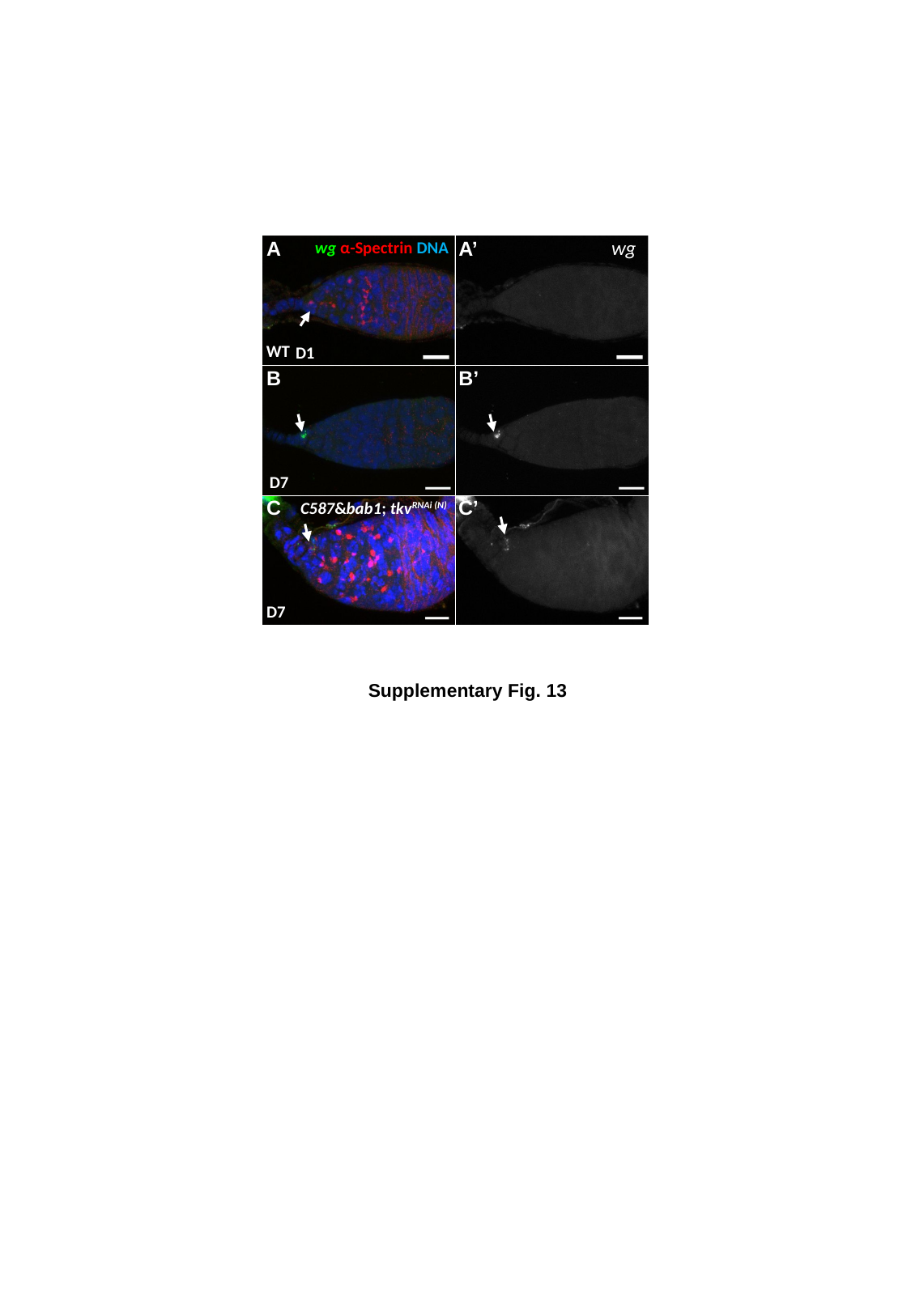

wg
A
A’
wg α-Spectrin DNA
WT
D1
B
B’
D7
C
C’
C587&bab1; tkvRNAi (N)
D7
Supplementary Fig. 13

## Slide 14
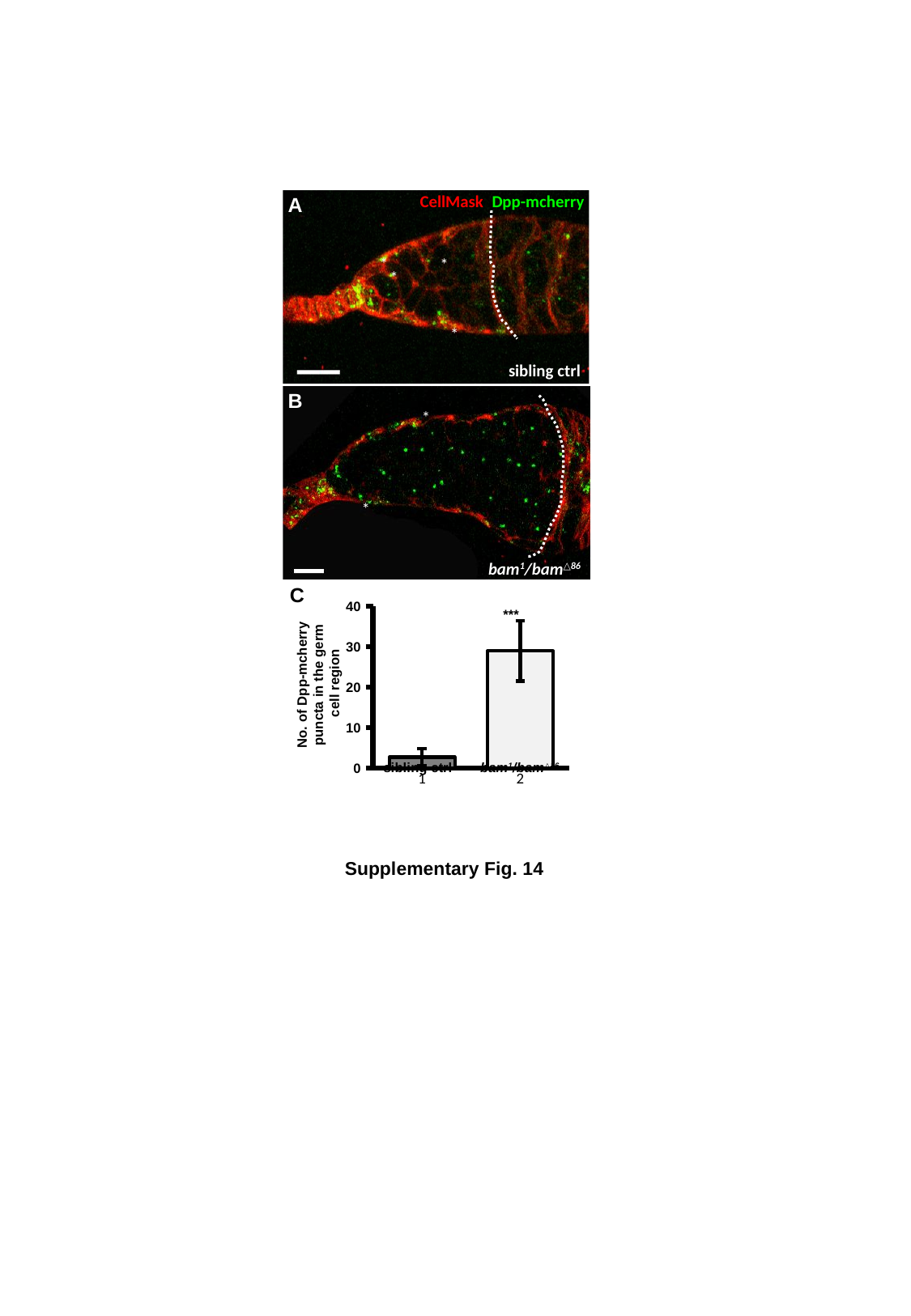

Dpp-mcherry
CellMask
A
*
*
*
*
sibling ctrl
B
*
*
bam1/bam△86
C
### Chart
| Category | |
|---|---|***
No. of Dpp-mcherry
puncta in the germ
cell region
sibling ctrl
bam1/bam△86
Supplementary Fig. 14
