# Supplementary material for: Canonical Wnt Signaling Promotes Formation of Somatic Permeability Barrier for Proper Germ Cell Differentiation
Source: Front Cell Dev Biol. 2022 Apr 19;10:877047. doi: 10.3389/fcell.2022.877047 (PMC9062081; doi:10.3389/fcell.2022.877047)
Supplement: Supplementary file 3 [file Table2.docx]

| **Supplementary Table 2. Primer sequences used in this study** | | |
| --- | --- | --- |
| **Primers used for qRT-PCR** | |  |
|  | **Primers used for *wg* and *fz3* fragments** |  |
| *wg* | **F** 5’-GGCTCGAAGACCTTGTCTATC-3’ |  |
|  | **R** 5’-ATGCAAGCAGTTCAAGCAGTG-3’ |  |
| *frz3* | **F** 5’-GCCAAACTGGTGCCACTAA-3’ |  |
|  | **R** 5’-ATTTAGGTGACACTATAGCAGGTAGAAGCACAGGGCAA-3’ |  |
| **Primers used for qRT-PCR after ChIP** | | |
| *cycB3*-P1  (-1313 to -1462) | **F** 5’- ATTGCTCCGGCCAATGAGTC -3′ |  |
|  | **R** 5’- ACATACTCGTCGTCCGCAAA -3′ |  |
| *cycB3*-P2  (-1235 to -1345) | **F** 5’- AACAAGTCCCTGTTTGCGGA -3′ |  |
|  | **R** 5’- CATGCCGAACAAACGGAACA -3′ |  |
| *cycB3*-CDS1  (1449 to 1569) | **F** 5′- ACTGTTCATGGCACTGCGTA -3′ |  |
|  | **R** 5′- CGCGGTCACAATTTCAGCAA -3′ |  |
| *cycB3-*CDS2  (1500 to 1636) | **F** 5′- GACGTGGACATCGACGCTTA -3′ |  |
|  | **R** 5′- TGTGCGAGTACTTGTTCCGT -3′ |  |
| *rp49* | **F** 5’-TCCGCCCAGCATACAGGC-3’ |  |
|  | **R** 5’-CAATCCTCGTTGGCACTCACC-3’ |  |

F, forward primer; R, reverse primer; P, promoter; CDS, coding sequence.

Numbers in parentheses indicate the nucleotide position of the *cycB3* fragment amplified by the indicated primer set.

ATTTAGGTGACACTATAG is the sequence of the SP6 promoter; fragments amplified by primers bearing the SP6 promoter were used to generate DNA templates for *in vitro* transcription.

qRT-PCR for *fz3* and *RPL19* was performed using the TaqMan system; qRT-PCR after ChIP was performed with the SYBR green system.
